# Supplementary material for: The impact of COVID-19 vaccination in the US: Averted burden of SARS-COV-2-related cases, hospitalizations and deaths
Source: PLoS One. 2023 Apr 25;18(4):e0275699. doi: 10.1371/journal.pone.0275699 (PMC10129007; doi:10.1371/journal.pone.0275699)
Supplement: S1 Text — (DOCX) [file pone.0275699.s001.docx]

**S1 Text. Additional model details**

**Model description**

We use a county-scale metapopulation model with Susceptible-Exposed-Infectious-Recovered (SEIR) structure to simulate the transmission of COVID-19 in the United States. This model has been used to in a number of recent publications [1-3], and was first described in Pei & Shaman [4].

We model the transmission dynamics of COVID-19 separately for day and night periods, reflecting the daytime movement of individuals, who return home for the night. Transmission is represented as a discrete Markov process during both day and night times. The daytime transmission lasts for $dt_{1}$ day and the nighttime transmission $dt_{2}$ day ($dt_{1}+dt_{2}=1)$. We assume daytime transmission lasts for 8 hours and nighttime transmission lasts for 16 hours, i.e., $dt_{1}=1/3$ and $dt_{2}=2/3$. The transmission dynamics are depicted by the following equations.

Daytime transmission:

$$S_{ij}\left( t+dt_{1} \right)=S_{ij}\left( t \right)-\frac{\beta S_{ij}\left( t \right)\sum_{k} I_{ki}^{r}\left( t \right)}{N_{i}^{D}\left( t \right)}dt_{1}-\frac{\mu\beta S_{ij}\left( t \right)\sum_{k} I_{ik}^{u}\left( t \right)}{N_{i}^{D}\left( t \right)}dt_{1}+\theta dt_{1}\frac{N_{ij}-I_{ij}^{r}\left( t \right)}{N_{i}^{D}\left( t \right)}\sum_{k\neq i} \frac{\bar{N}_{ik}\sum_{l} S_{kl}\left( t \right)}{N_{k}^{D}\left( t \right)-\sum_{l} I_{lk}^{r}\left( t \right)}-\theta dt_{1}\frac{S_{ij}\left( t \right)}{N_{i}^{D}\left( t \right)-\sum_{l} I_{li}^{r}\left( t \right)}\sum_{k\neq i} \bar{N}_{ki} (1)$$

$$E_{ij}\left( t+dt_{1} \right)=E_{ij}\left( t \right)+\frac{\beta S_{ij}(t)\sum_{k} I_{ki}^{r}(t)}{N_{i}^{D}(t)}dt_{1}+\frac{\mu\beta S_{ij}(t)\sum_{k} I_{ik}^{u}(t)}{N_{i}^{D}(t)}dt_{1}-\frac{E_{ij}(t)}{Z}dt_{1}+\theta dt_{1}\frac{N_{ij}-I_{ij}^{r}(t)}{N_{i}^{D}(t)}\sum_{k\neq i} \frac{\bar{N}_{ik}\sum_{l} E_{kl}(t)}{N_{k}^{D}(t)-\sum_{l} I_{lk}^{r}(t)}-\theta dt_{1}\frac{E_{ij}(t)}{N_{i}^{D}(t)-\sum_{l} I_{li}^{r}(t)}\sum_{k\neq i} \bar{N}_{ki} (2)$$

$$I_{ij}^{r}\left( t+dt_{1} \right)=I_{ij}^{r}\left( t \right)+\alpha\frac{E_{ij}(t)}{Z}dt_{1}-\frac{I_{ij}^{r}(t)}{D}dt_{1} (3)$$

$$I_{ij}^{u}\left( t+dt_{1} \right)=I_{ij}^{u}\left( t \right)+\left( 1-\alpha\right)\frac{E_{ij}(t)}{Z}dt_{1}-\frac{I_{ij}^{u}\left( t \right)}{D}dt_{1}+\theta dt_{1}\frac{N_{ij}-I_{ij}^{r}\left( t \right)}{N_{i}^{D}\left( t \right)}\sum_{k\neq i} \frac{\bar{N}_{ik}\sum_{l} I_{kl}^{u}\left( t \right)}{N_{k}^{D}\left( t \right)-\sum_{l} I_{lk}^{r}\left( t \right)}-\theta dt_{1}\frac{I_{ij}^{u}(t)}{N_{i}^{D}(t)-\sum_{l} I_{li}^{r}(t)}\sum_{k\neq i} \bar{N}_{ki} (4)$$

$$N_{i}^{D}(t)=N_{ii}+\sum_{k\neq i} I_{ki}^{r}(t)+\sum_{k\neq i} (N_{ik}-I_{ik}^{r}(t)) (5)$$

Nighttime transmission:

$$S_{ij}^{'}\left( t+1 \right)=S_{ij}\left( t+dt_{1} \right)-\frac{\beta S_{ij}\left( t+dt_{1} \right)\sum_{k} I_{kj}^{r}\left( t+dt_{1} \right)}{N_{j}^{N}}dt_{2}-\frac{\mu\beta S_{ij}\left( t+dt_{1} \right)\sum_{k} I_{kj}^{u}\left( t+dt_{1} \right)}{N_{j}^{N}}dt_{2}+\theta dt_{2}\frac{N_{ij}}{N_{j}^{N}}\sum_{k\neq j} \frac{\bar{N}_{jk}\sum_{l} S_{lk}\left( t+dt_{1} \right)}{N_{k}^{N}-\sum_{l} I_{lk}^{r}\left( t+dt_{1} \right)}-\theta dt_{2}\frac{S_{ij}\left( t+dt_{1} \right)}{N_{j}^{N}-\sum_{k} I_{kj}^{r}\left( t+dt_{1} \right)}\sum_{k\neq j} \bar{N}_{kj} (6)$$

$$E_{ij}\left( t+1 \right)=E_{ij}\left( t+dt_{1} \right)+\frac{\beta S_{ij}(t+dt_{1})\sum_{k} I_{kj}^{r}(t+dt_{1})}{N_{j}^{N}}dt_{2}+\frac{\mu\beta S_{ij}(t+dt_{1})\sum_{k} I_{kj}^{u}(t+dt_{1})}{N_{j}^{N}}dt_{2}-\frac{E_{ij}\left( t+dt_{1} \right)}{Z}dt_{2}+\theta dt_{2}\frac{N_{ij}}{N_{j}^{N}}\sum_{k\neq j} \frac{\bar{N}_{jk}\sum_{l} E_{lk}\left( t+dt_{1} \right)}{N_{k}^{N}-\sum_{l} I_{lk}^{r}\left( t+dt_{1} \right)}-\theta dt_{2}\frac{E_{ij}(t+dt_{1})}{N_{j}^{N}-\sum_{k} I_{kj}^{r}(t+dt_{1})}\sum_{k\neq j} \bar{N}_{kj} (7)$$

$$I_{ij}^{r}\left( t+1 \right)=I_{ij}^{r}\left( t+dt_{1} \right)+\alpha\frac{E_{ij}(t+dt_{1})}{Z}dt_{2}-\frac{I_{ij}^{r}\left( t+dt_{1} \right)}{D}dt_{2} (8)$$

$$I_{ij}^{u}\left( t+1 \right)=I_{ij}^{u}\left( t+dt_{1} \right)+\left( 1-\alpha\right)\frac{E_{ij}\left( t+dt_{1} \right)}{Z}dt_{2}-\frac{I_{ij}^{u}\left( t+dt_{1} \right)}{D}dt_{2}+\theta dt_{2}\frac{N_{ij}}{N_{j}^{N}}\sum_{k\neq j} \frac{\bar{N}_{jk}\sum_{l} I_{lk}^{u}\left( t+dt_{1} \right)}{N_{k}^{N}-\sum_{l} I_{lk}^{r}\left( t+dt_{1} \right)}-\theta dt_{2}\frac{I_{ij}^{u}\left( t+dt_{1} \right)}{N_{j}^{N}-\sum_{k} I_{kj}^{r}\left( t+dt_{1} \right)}\sum_{k\neq j} \bar{N}_{kj} (9)$$

$$N_{i}^{N}=\sum_{k} N_{ki} (10)$$

Where $S_{ij}$, $E_{ij}$, $I_{ij}^{r}$, $I_{ij}^{u}$ and $N_{ij}$ are the susceptible, exposed, reported infected, unreported infected and total populations in the subpopulation commuting from county $j$ to county $i$ ($i\leftarrow j$). $\beta$ is the transmission rate of reported infections; $\mu$ is the relative transmissibility of unreported infections; $Z$ is the average latency period (from infection to contagiousness); $D$ is the average duration of contagiousness; $\alpha$ is the fraction of documented infections; $\theta$ is a multiplicative factor adjusting random movement; $\bar{N}_{ij}=(N_{ij}+N_{ji})/2$ is the average number of commuters between counties $i$ and $j$; and $N_{i}^{D}$ and $N_{i}^{N}$ are the daytime and nighttime populations of county $i$. Equations 1 through 10 are integrated using a Poisson process, introducing stochasticity into the transmission dynamics.

Following the integration, we simulate vaccination by moving vaccinated susceptible individuals from the susceptible compartment to the recovered compartment as follows:

$$S_{ij}\left( t+1 \right)=S_{ij}^{'}\left( t+1 \right)-\frac{S_{ij}\left( t-t_{vax} \right)}{N_{ij}}{\frac{N_{ij}}{N_{s}}V}_{s}\left( t-t_{vax} \right)* v_{eff}$$

Where V_s_(t) is the number of individuals vaccinated at time *t* in the state *s* containing county *j*. The vaccine takes effect *t_vax_* days after administration, and protects the vaccinated susceptible individuals with probability *v_eff_.* We assume that the number of vaccines are distributed across each state’s subpopulations proportionally to the subpopulation size, and that individuals are vaccinated independently of their prior infection status (ie. Individuals in the susceptible compartment are no more or less likely to be vaccinated than members of other compartments).

Population movement between US counties is represented using commuting data publicly available from the US Census Bureau [5]. We modify the pre-pandemic commuting levels using estimates of relative changes in inter-county visits derived from SafeGraph, a data company that aggregates anonymized location data from numerous applications in order to provide insights about physical places, via the SafeGraph Community. To enhance privacy, SafeGraph excludes census block group information if fewer than two devices visited an establishment in a month from a given census block group [6]. We also model random movement between counties at a rate proportional the numbers of inter-county commuters.

To account for delay between a person becoming infectious with Covid-19 and being recorded as a confirmed case, we mapped simulated reported infections to confirmed cases using a separate observational delay model. In this delay model, we account for the time interval between a person transitioning from latent to contagious (i.e. *E 🡪* $I_{i}^{r}$) and observational confirmation of that individual infection.

**Model calibration**

We calibrated the transmission model using daily county-level COVID-19 compiled by Johns Hopkins Center for Systems Science and Engineering [7]. These observational data were used to infer state variables and time-varying, county-specific $\alpha$ and $\beta$parameters values using an ensemble adjustment Kalman filter (EAKF) , a data assimilation method originally developed for weather forecasting [8], and more recently applied to successfully infer epidemiological parameters for a number of infectious diseases including COVID-19 [2], influenza [9], and RSV[10].

We ran an ensemble of 100 model simulations, in which parameters and state variables randomly initialized and iteratively optimized by the EAKF following each daily observation in a prediction-update cycle. In the prediction step, state variables are propagated forward in time by the disease transmission model. In the update step, the filter algorithms adjust ensemble members in order to better match the observation. The updates applied to unobserved state variables and parameters are linear mappings from the update applied to the observed variable based on the prior ensemble covariance between the observed and unobserved variables. The update step is computed such that the posterior ensemble mean and variance match the mean and variance predicted by Bayes theorem, assuming a Gaussian distribution [8].

The time- and spatially-varying ascertainment rates and transmission rates are estimated by the model. In order to reduce the number of parameters being estimated, parameter values for disease-related parameters $\mu$, $Z$, $D$ and the mobility factor $\theta$ are assigned are fixed, as shown in Table S2.

The time-varying reproductive number Rt, is computed for county *c* and time *t* as:

$R_{t}=\beta\left( c,t \right)D[\alpha\left( c,t \right)+\mu(1-\alpha(c,t))]$

The changes in *R_t_* applied in Counterfactual Scenarios 2 and 3 are applied as changes in $\beta\left( c,t \right)$.

**S1 Table. Model parameters**

| Model parameters | description | Spatial granularity | Value | Reference |
| --- | --- | --- | --- | --- |
| $\alpha$ | Ascertainment rate | County | Estimated | n/a |
| $\beta$ | Transmission rate | County | Estimated | n/a |
| Z | Incubation period | US | 3.63 (95% CI: 3.41 – 3.86) | [4] |
| *D* | Duration of infectiousness | US | 3.50 (3.14 – 3.83) | [4] |
| $\mu$ | Relative infectiousness of asymptomatic infection | US | 0.61 (0.56 – 0.68) | [4] |
| $\theta$ | Mobility factor | Fixed | 0.15 (0.12 – 0.17) | [4] |
| *V_eff_* | Vaccine effectiveness | Fixed | 90% | [11-13] |

**References**

1. Pei S, Kandula S, Shaman J. Differential effects of intervention timing on COVID-19 spread in the United States. Science advances. 2020;6(49):eabd6370.

2. Pei S, Yamana TK, Kandula S, Galanti M, Shaman J. Burden and characteristics of COVID-19 in the United States during 2020. Nature. 2021;598(7880):338-41.

3. Pei S, Dahl KA, Yamana TK, Licker R, Shaman J. Compound risks of hurricane evacuation amid the COVID‐19 pandemic in the United States. GeoHealth. 2020;4(12):e2020GH000319.

4. Pei S, Shaman J. Initial simulation of SARS-CoV2 spread and intervention effects in the continental US. MedRxiv. 2020.

5. US Census Bureau. 2020. Available from: <https://www.census.gov/topics/employment/commuting.html>.

6. Safe Graph. 2020. Available from: safegraph.com.

7. CSSE J. COVID-19 Data Repository by the Center for Systems Science and Engineering (CSSE) at Johns Hopkins University 2020 [cited 2021 November 15]. Available from: <https://github.com/CSSEGISandData/COVID-19>.

8. Anderson JL. An ensemble adjustment Kalman filter for data assimilation. Monthly weather review. 2001;129(12):2884-903.

9. Yang W, Lipsitch M, Shaman J. Inference of seasonal and pandemic influenza transmission dynamics. Proceedings of the National Academy of Sciences. 2015;112(9):2723-8.

10. Reis J, Shaman J. Retrospective parameter estimation and forecast of respiratory syncytial virus in the United States. PLoS computational biology. 2016;12(10):e1005133.

11. Polack FP, Thomas SJ, Kitchin N, Absalon J, Gurtman A, Lockhart S, et al. Safety and Efficacy of the BNT162b2 mRNA Covid-19 Vaccine. New England Journal of Medicine. 2020;383(27):2603-15. doi: 10.1056/NEJMoa2034577. PubMed PMID: 33301246.

12. Baden LR, El Sahly HM, Essink B, Kotloff K, Frey S, Novak R, et al. Efficacy and Safety of the mRNA-1273 SARS-CoV-2 Vaccine. New England Journal of Medicine. 2020;384(5):403-16. doi: 10.1056/NEJMoa2035389. PubMed PMID: 33378609.

13. Sadoff J, Gray G, Vandebosch A, Cárdenas V, Shukarev G, Grinsztejn B, et al. Safety and Efficacy of Single-Dose Ad26.COV2.S Vaccine against Covid-19. New England Journal of Medicine. 2021;384(23):2187-201. doi: 10.1056/NEJMoa2101544. PubMed PMID: 33882225.
