# Supplementary figures and images for: The impact of COVID-19 vaccination in the US: Averted burden of SARS-COV-2-related cases, hospitalizations and deaths
Source: PLoS One. 2023 Apr 25;18(4):e0275699. doi: 10.1371/journal.pone.0275699 (PMC10129007; doi:10.1371/journal.pone.0275699)

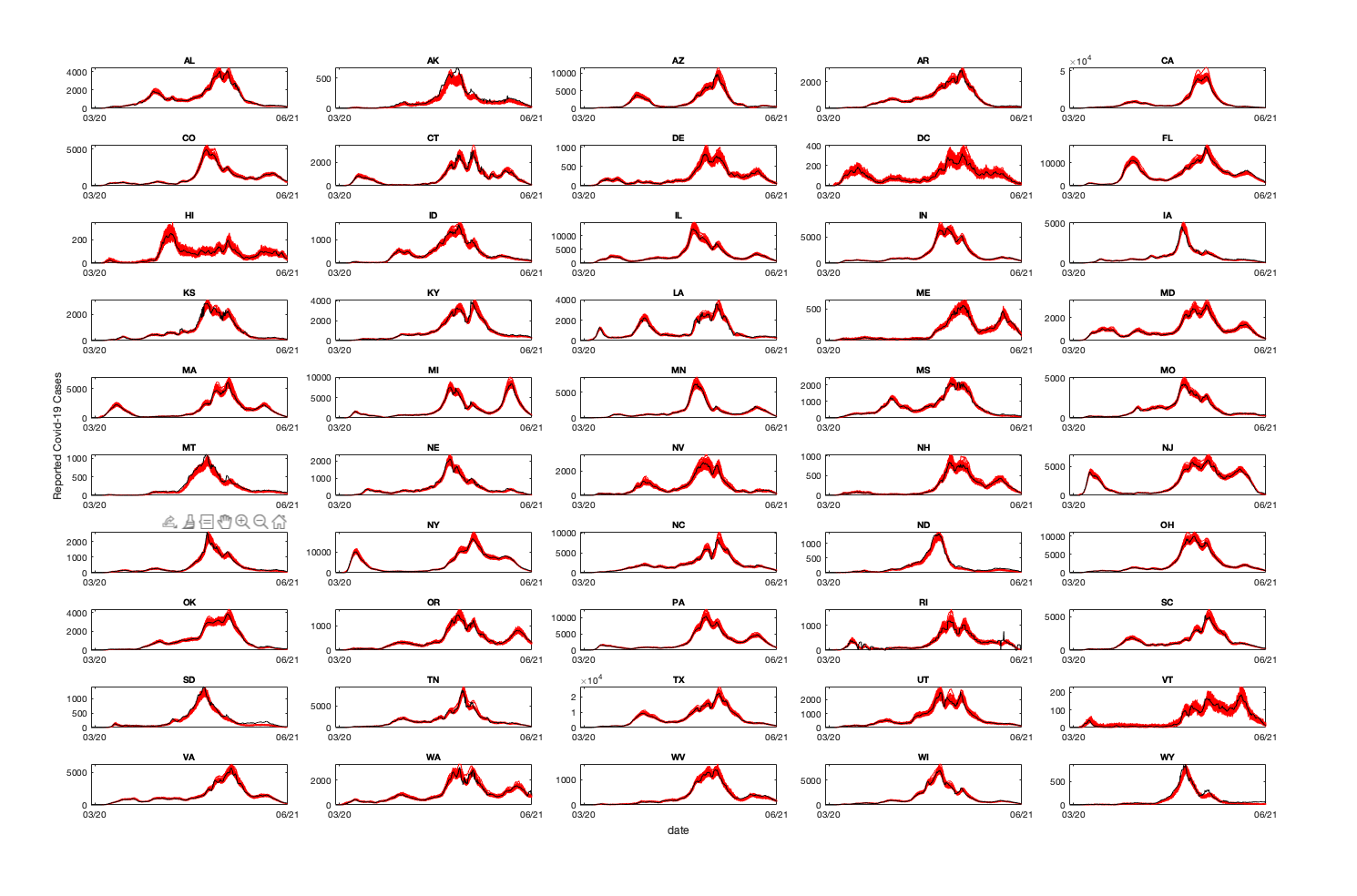

Supplement: S1 Fig — Observed data are shown in black (7-day backwards looking moving average) and fitted model results are in red. (TIF) [file pone.0275699.s002.tif]

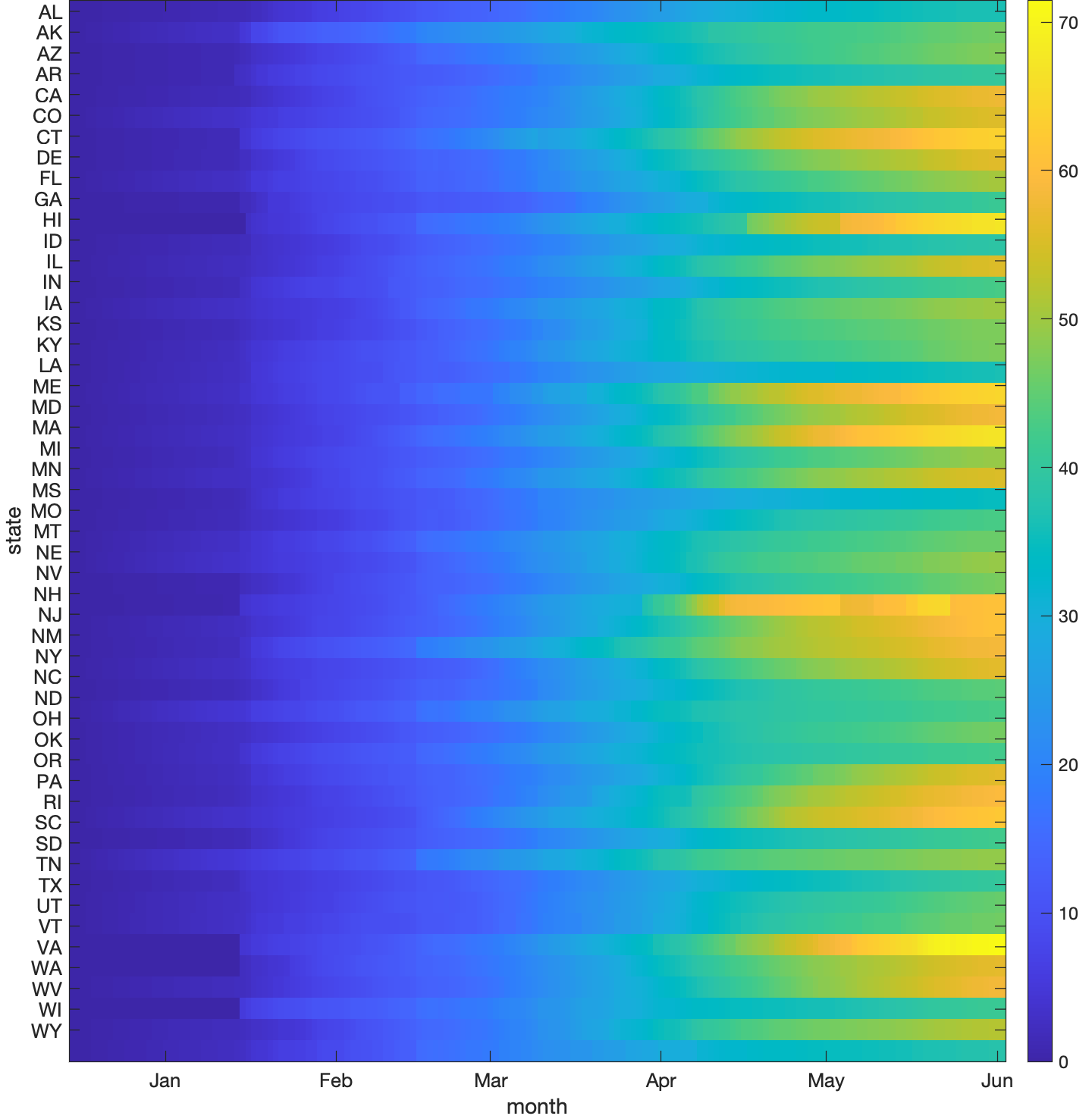

Supplement: S2 Fig — The vaccination rate as a percentage of total state population is shown as indicated in the color bar. (TIF) [file pone.0275699.s003.tif]
