# Supplementary material for: The impact of COVID-19 vaccination in the US: Averted burden of SARS-COV-2-related cases, hospitalizations and deaths
Source: PLoS One. 2023 Apr 25;18(4):e0275699. doi: 10.1371/journal.pone.0275699 (PMC10129007; doi:10.1371/journal.pone.0275699)
Supplement: Attachment — Submitted filename: Response to reviewers_clean.docx [file pone.0275699.s005.docx]

We thank the reviewers and the editor for their constructive feedback. We have addressed each of the comments in the manuscript, as well as in the responses below.

**Reviewer #1:** Vaccination programs contributed to preventing the number of infected with SARS-CoV-2, hospitalizations and deaths related to COVID-19 worldwide. Quantifying the averted those burdens is crucial to evaluate the impact of the vaccination and decision-making at the time. Using the meta-population model, the authors tried to estimate the numbers of prevented SARS-CoV-2-related cases, hospitalizations and deaths attributable to vaccination in the US. Because the authors applied the model previously published in a few scientific journals, I believe the model could be robust even in the current context of the manuscript. However, I have some comments on the manuscript, so I would like the authors to consider them carefully.


1) In terms of the characteristics of the effectiveness of the vaccination, could the authors clarify it? The effectiveness would be divided into two: direct and indirect. I presume that the authors estimated the direct effect of vaccination because they did not take into account the reduction of the transmission rate of vaccinated people. I would be grateful if the authors discussed such effectiveness using the cited references (e.g., Israel’s study explored the direct effect but not the indirect one).

Thank you for raising this point – we have clarified this issue in the revised manuscript. We assumed vaccination to be fully protective against infection, so our analysis does include the indirect effects of vaccination. That is, because vaccinated individuals cannot be infected, they also cannot transmit the disease to others, thereby adding a level of protection to unvaccinated individuals. We have clarified this in the manuscript. We have also specified in the discussion which of the cited references consider direct vs indirect effects.

2) Please provide the fitted baseline scenarios for counties or states; otherwise, it is difficult to judge the scientific validation of the calibrated mode.

Thank you for this suggestion - the fitted baseline scenarios for each state have been added.

3) I would recommend that the authors provide information on the model even though the model was previously well described elsewhere. For example, how did the authors cooperate vaccination into the model? Unfortunately, I could not understand how they were considered even though I read the Supplementary Material. Please share the equation.

We expanded the model description, which now includes the equation used to simulate vaccinate to the model description and is found in the Supplementary Material. We have also included a table of model parameters (Table S1).

4) I agree with the advantages of the meta-population model that could capture the various characteristics of county or state-specific. However, some measures, e.g., CHR and CFR, are very important to consider the discrepancy between age groups. Therefore, please validate the usage of averages of such measures by showing the comparison between observed values and estimates of the hospitalizations and deaths from baseline scenarios, for example.

We have added a comparison of observed and estimated hospitalizations and deaths:

“Between December 14 and June 3, 2021, the baseline model estimated 16.1 million (95% CrI 15.1 – 18.3 million) total cases, 1.4 million (95% CrI 1.3 – 1.6 million) hospitalizations, and 246.7 thousand (95% CrI 230.4 – 279.6 thousand) deaths across the United States. These estimates were consistent with the 16.7 million cases, 1.3 million hospitalizations, and 250 thousand deaths reported in the JHU CSSE and HHS hospitalization datasets [1, 2].”

Additionally, we have added discussion on the impact of the age discrepancies.

5) I do not understand why the authors calculated averted hospitalization costs. Please provide more insights obtained from the analyses in the discussion.

Thank you for your comment. We have further expanded the Discussion with the following:

Additionally, our study sought to quantify the cost savings associated with vaccine-preventable severe disease (i.e. hospitalizations). The COVID-19 pandemic has challenged the capacity of hospitals and strained hospital and health system finances [3]. In Scenario 3, we estimated that, in the first six months of rollout of the COVID-19 Vaccination program, the hospitalization cost savings were $17.3 billion. More conservatively, Scenario 1 estimated cost savings of $7.0 billion. While these numbers are not adjusted for geographic differences, they provide a ballpark estimate that is in line with a previously published analysis, which estimated $13.8 billion of costs from preventable COVID-19 hospitalizations from June through November 2021 [4]. Our analyses show that the benefits of vaccination due to reduced hospitalizations translated into cost-savings in the billions of dollars. The broad availability of COVID-19 vaccines brought wide-ranging benefits from both a public health and economic perspective. Vaccination may also lessen other societal impacts associated with the pandemic (e.g. work productivity loss). The total economic impact may therefore be even greater than reported, and further studies elucidating those impacts are warranted.

6) In the result, the authors showed that 74.1% were susceptible on 14 December 2020. It would be great if the authors compared this value and observed one, possibly from serological surveys, in order to validate the baseline model.

The model estimates of susceptibility have previously been validated with serological data [5]. Specifically, estimates of cumulative infections during 2020, generated by this model when coupled with inference approaches, were compared and validated with estimates of seroprevalence derived from serological surveys collected on multiple dates and for multiple locations in the US. These external serological data provided strong validation of the model estimates of cumulative infections and thus population susceptibility.

7) Please provide results shown in Table 1 for each county or state as tables or figures (cumulative) in the result or Supplementary Material.

A table presenting the mean number of averted cases, hospitalizations and deaths in each location has been added to the Supplementary Material.

8) Although the authors mentioned some results (i.e. averted cases, hospitalizations and deaths) estimated from previously published studies, there are no mentions of the methodology differences. For the direct effect of the vaccination, averted cases and hospitalizations can be calculated by the difference in incidences between unvaccinated and vaccinated. Why did the authors use the meta-population model? If the interactions between counties or states, why do not explore the insights (e.g. difference between states) further? Those points need to be described more in the manuscript.

We used the metapopulation model for this study as it has been extensively validated and used both for research and operational purposes over the course of the pandemic. In the revised manuscript, we have expanded our presentation of state-level results.

**Reviewer #2:** This paper aims at caracterizing the averted covid-19 burden from vaccination in the first 6 months of the campaign in the US. It's a short paper, to the point, with a simple application of an already developed model. While the paper is suitable for publication in PLoS One, I think there are some barriers to have it published as it is.

1) Mainly, a lot of details are lacking in understanding the paper. Mainly the methods are not described fully (but available from other papers), and some diagnosis figures (posterior predictive checks or equivalent, vaccination baseline scenario model fit ...) are missing.

Thank you for pointing out this oversight. We have added a figure showing baseline scenarios for each state. We have also added more information about the model structure and parameterization.

**Some other remarks:**

2) One question: why model at county scale but only present results at national or state-scale ?

While our model simulates Covid transmission dynamics at the county scale, we use state level data to inform our CHR, CFR and vaccination rates. We chose to present results at state scale to avoid introducing further uncertainty into the model by disaggregating these state-level data.

3) « We modeled the vaccine as producing direct effects only » but with susceptible depletion, aren’t some indirect effects also taken into account in this model ?

Thank you for raising this point – we should not have used the phrase ‘direct effects’ here, and have clarified this in the manuscript.

We assumed vaccination to be fully protective against infection, so our analysis does include the indirect effects of vaccination. That is, because vaccinated individuals cannot be infected, they also cannot transmit the disease to others, thereby adding a level of protection to unvaccinated individuals.

4) A lot of simplifying assumptions are made but the discussion does not detail enough their possible impact on concusion (especially focusing only on the first six month without omicron, not using an age-stratified model, no discussion on delayed vs averted death, )

We have added the following discussion on delayed vs averted deaths:

“… the establishment of the more virulent Delta and immune-evading Omicron variants have made estimation of vaccine effects more challenging. These later phases of the pandemic driven by new variants led to tens of millions of Covid-19 infections. We are not able to say definitively whether averted cases, hospitalizations and deaths quantified in this analysis were truly averted or merely delayed. Nevertheless, it can be argued that these early averted cases were crucial, as this period was prior to the widespread availability of antiviral medication and a time with substantially lower population immunity against severe outcomes.”

We have also discussed the impact of non-age stratified model

“These assumptions may have led to biases in our results, as the risk of both hospitalization and death following COVID-19 infection are known to increase with age. Since our CHR and CFRs were calculated based on population-level averages, we likely underestimated the number of averted hospitalizations and deaths in the first few months of the analysis when vaccine uptake was concentrated in populations most at risk of severe outcomes.”

5) "All 3 scenarios show that vaccination benefits were limited during the early months of vaccine rollout" This might an artifact from the age-prioritization of covid-19 no ?

We believe this finding is explained due to the relatively small proportion of vaccinated individuals, as well as the overall trend of declining transmission during that time.

6) What makes the model finding so different from other modeling studies cited in the conclusion ?.

Our findings are generally in agreement with previous studies cited in the manuscript. We have expanded our discussion of the differences in our methodological approach.

Our study also adds to the existing literature by considering 3 counterfactual scenarios, all without vaccinations, but with varying R_t,_ that mimic different possible population responses to disease spread in the absence of a vaccine.

The paper is not standalone:
7) "and its [the model] full details are described in Pei & Shaman [4]."
8) "Parameter values for μ, Z, D and θ are assigned according to the values inferred in Pei and Shaman [4]."

We have removed the dependence Pei & Shaman by providing a full model description and a table of model parameters (Table S1).

9) Figure S1 is refered nowhere in the main text nor in the SI text.

We added the reference in the methods.

10) So with the addition that the code is not shared yet, nor are figures of model fits, so it's really hard to provide a proper review of the paper. Especially as this study is sponsored by Pfizer, there should be a strong justification of the conclusions from the methods, which I cannot judge due to the lack of insight.

We hope that the revised materials provide confidence in the study methods and conclusions.

References

1.	CSSE J. COVID-19 Data Repository by the Center for Systems Science and Engineering (CSSE) at Johns Hopkins University 2020 [cited 2021 November 15]. Available from: <https://github.com/CSSEGISandData/COVID-19>.

2.	Health UDo, Services H. COVID-19 reported patient impact and hospital capacity by facility 2020 [cited 2021 November 15]. Available from: <https://healthdata.gov/Hospital/COVID-19-Reported-Patient-Impact-and-Hospital-Capa/g62h-syeh>.

3.	Association AH. Massive growth in expenses and rising inflation fuel continued financial challenges for America’s hospitals and health systems. Cost of Caring Report Published April. 2022.

4.	Amin K, Cox C. Unvaccinated COVID-19 hospitalizations cost billions of dollars. Health System Tracker Available at: <https://bit> ly/3GTceUq [cited 2021, Dec 10]. 2022.

5.	Pei S, Yamana TK, Kandula S, Galanti M, Shaman J. Burden and characteristics of COVID-19 in the United States during 2020. Nature. 2021;598(7880):338-41.
