# Supplementary material for: The impact of COVID-19 vaccination in the US: Averted burden of SARS-COV-2-related cases, hospitalizations and deaths
Source: PLoS One. 2023 Apr 25;18(4):e0275699. doi: 10.1371/journal.pone.0275699 (PMC10129007; doi:10.1371/journal.pone.0275699)
Supplement: Attachment — Submitted filename: response to reviewers.docx [file pone.0275699.s006.docx]

We thank the editor and reviewers for your time and comments. We have addressed each of the comments below, and in the manuscript.

*Editor:*

*The manuscript has been well revised overall. However, I concur with Reviewer 1's comment regarding potential biases in CFR and CHR. Such naively calculated CFR and CHR may have been underestimated if the time delay from infection (or confirmation) to death (or hospitalization) was not fully taken into consideration, especially if the epidemic size exponentially increased in the corresponding period. Thus, I would like to strongly recommend that authors reestimate the CFR and CHR while taking the time delay distribution into consideration.*

Thank you for raising this issue. We fully agree that the delays between case reports and the corresponding hospitalizations and deaths are important. We did in fact apply delays, but this was not clearly explained in our original manuscript. We have edited the manuscript to be more precise.

*Reviewer #1: I am concerned about CHR and CFR. When the authors estimated CFR as dividing cumulative deaths reported between Aug 1 to Dec 14 2020 by cumulative cases infected during the same period, it may lead to an underestimate due to the right censoring issue of reported deaths (reporting delay between infection and death), especially in the case the magnitude of infections is large in the late period.*

Thank you for raising this issue. We fully agree that the delays between case reports and the corresponding hospitalizations and deaths are important. We did in fact apply delays, but this was not clearly explained in our original manuscript. We have edited the manuscript to be more precise.

*Reviewer #2: Thanks for including the code and for making the paper standalone. I appreciated the comments added in the description.
I do not have any comment that would require another review. Just two things, not mandatory
- l106: . "We modeled the vaccine as providing 90% effectiveness against infection"
I think "and transmission" should be added there to make sure there isn't any misunderstanding. (perhaps that could be also mentionned in discussion).*

Thank you for your comments. We have added ‘and transmission’ to lines 107 and 108 to avoid any misinterpretation.

*- fig S2: any tentatitive explanation of why Rt is higher in winter than in summer would be welcome. I do think this figures should be in the main text (with another pannel being vaccines uptake ?).*

We moved the figure to the main text. We provide some possible explanations for temporal changes in Rt in the discussion (eg population behavior, new variant, Lines 387-390). While we’re very interested in other possible factors, this does not fall within the scope of this study.
